# Peritraumatic Stress From a Disaster Increases Risk for Onset of Chronic Diseases Among Older Adults

**DOI:** 10.1093/geroni/igab052

**Published:** 2022-01-01

**Authors:** Laura P Sands, Quyen Do, Pang Du, Rachel Pruchno

**Affiliations:** 1 Center for Gerontology, Virginia Tech, Blacksburg, Virginia, USA; 2 Department of Statistics, Virginia Tech, Blacksburg, Virginia,USA; 3 New Jersey Institute for Successful Aging, Rowan University School of Osteopathic Medicine, Stratford, New Jersey, USA

**Keywords:** Disaster, Hospital, Hurricane Sandy

## Abstract

**Background and Objectives:**

Our understanding of the impact of disaster exposure on the physical health of older adults is largely based on hospital admissions for acute illnesses in the weeks following a disaster. Studies of longer-term outcomes have centered primarily on mental health. Missing have been studies examining whether exposure to disaster increases the risk for the onset of chronic diseases. We examined the extent to which 2 indicators of disaster exposure (geographic exposure and peritraumatic stress) were associated with new onset of cardiovascular disease, diabetes, arthritis, and lung disease to improve our understanding of the long-term physical health consequences of disaster exposure.

**Research Design and Methods:**

We linked self-reported data collected prior to and following Hurricane Sandy from a longitudinal panel study with Medicare data to assess time to new onset of chronic diseases in the 4 years after the hurricane.

**Results:**

We found that older adults who reported high levels of peritraumatic stress from Hurricane Sandy had more than twice the risk of experiencing a new diagnosis of lung disease, diabetes, and arthritis in the 4 years after the hurricane compared to older adults who did not experience high levels of peritraumatic stress. Geographic proximity to the hurricane was not associated with these outcomes. Analyses controlled for known risk factors for the onset of chronic diseases, including demographic, psychosocial, and health risks.

**Discussion and Implications:**

Findings reveal that physical health effects of disaster-related peritraumatic stress extend beyond the weeks and months after a disaster and include new onset of chronic diseases that are associated with loss of functioning and early mortality.

**Translational Significance:** Little is known about the impact of disaster exposure on older adults’ physical health in the years after a disaster. We found that older adults who experienced a lot of fear and distress during Hurricane Sandy were at significantly increased risk for new onset of chronic conditions including lung disease, diabetes, and arthritis in the 4 years after the hurricane. The findings suggest that older adults who experience a lot of disaster-related fear and distress may benefit from health monitoring and health education with the goal of preventing the onset of chronic conditions.

## Background and Objectives

Natural disasters such as hurricanes, earthquakes, wildfires, and tornados create sudden, unexpected challenges that have the potential to threaten the health and well-being of individuals. On October 29, 2012, Hurricane Sandy, the largest Atlantic coast hurricane, caused 70 million dollars in property damage as 8.5 million people lost power for weeks, 350 000 persons evacuated their homes, and 117 people died (1). While many older adults fare well during and after a disaster (2), those with chronic diseases, physical limitations, and few social resources are at higher risk for poor health outcomes after a disaster than those without these risks (3–6). Number of chronic diseases and physical limitations increases with age, particularly for those aged 65 years and older (7, 8). More chronic diseases and greater physical limitations are associated with hospitalization and mortality (9). Thus, it is not surprising that half of the deaths due to Hurricane Sandy were among those aged 65 and older (10), and rates of hospitalization increased significantly among older adults in the weeks and months after the disaster (11–14).

Although the immediate effects of natural disasters on physical health are well documented (2, 15), little is known about the long-term effects of natural disasters on the physical health of older people (3). A few studies found that disaster exposure is associated with declines in older adults’ physical functioning in the years after a disaster (16–18). Theoretical studies describe the impact of chronic diseases on functioning (19, 20), and multiple empirical studies provide robust evidence that chronic disease status increases the risk for functional decline (21–24). Yet to date, questions remain about whether disaster exposure increases older adults’ risk for onset chronic diseases, which in turn increase the risk of functional decline and mortality.

The increasing frequency and severity of disasters in the last 2 decades (15, 25), combined with emerging evidence that disaster exposure has long-term impacts on older adults’ physical functioning, signals the importance of determining how disaster exposure affects the onset of chronic diseases in the aftermath of a disaster. The purpose of this study is to examine whether disaster exposure is associated with new onset of chronic diseases in the years after a disaster. Findings will inform secondary preventions strategies (15) by identifying older adults who are at risk for postdisaster onset of new chronic diseases.

### Conceptual Framework

Stress occurs when an individual perceives that the demands of environmental stimuli exceed their capacity to address those demands (26). Stress triggers multisystem physiological responses (27) including dysregulation in neuroendocrine, metabolic, immune, and cardiovascular systems (28–30). These physiological responses can in turn lead to early biologic aging and disease (27). Expert reviews of physiological responses to stress provide robust evidence that stress is associated with the onset of chronic diseases (31), including cardiovascular disease (CVD) (32), diabetes (33), asthma (30), and arthritis (34). Yet, little is known about whether exposure to a natural disaster is associated with the onset of chronic diseases in the years following a disaster. There is, however, some evidence that disasters can have long-term effects on the physical health of older adults. For example, a study of persons exposed to both Hurricane Katrina and the Deep Horizon Oil Spill reported more symptoms of poor health such as wheezing, tightness in the chest, heart palpitations, and fatigue in the years after these disasters than persons exposed to only one of these events (35). In addition, studies have assessed physiologic markers of stress from a disaster (36, 37). For example, a study of relocated Hurricane Katrina survivors and matched controls showed that exposure to Hurricane Katrina was associated with higher interleukin 6 for those with posttraumatic stress disorder (PTSD), a commonly accepted marker of disaster-related stress (38). Another study found higher heart rates and blood pressure reactivity 20 months after the hurricane (39). Collectively, these studies suggest that stressors associated with disaster exposure may lead to stress-related physical health consequences.

When disaster strikes, people experience a multitude of stressors, including immediate physical danger, fear and distress, injury, loss of a friend or loved one, property damage, and loss of utilities (17, 40). Prior research found that people experiencing high levels of peritraumatic stress—feeling in immediate physical danger or experiencing fear and distress—have more depression (41, 42) and poorer physical functioning (17) than people not experiencing high levels of peritraumatic stress. In addition, a study that linked self-reported data and Medicare claims found that self-reports of fear and distress were associated with greater use of emergency department admissions (12) during the year after the disaster.

The relationship between geographic proximity to disaster and loss of physical functioning in the years following the disaster is less clear. One study of older adults exposed to the Great East Japan earthquake and tsunami found that geographic proximity to the disaster is associated with loss of physical functioning (16), and other study of older adults exposed to Hurricane Sandy found that geographic proximity to the disaster is not associated with loss of physical functioning (17). While some research finds that indicators of disaster exposure, including sustaining injuries, losing a loved one, experiencing property loss, and losing utilities are unrelated to physical decline (17), other research finds more complex relationships. A study of older adults who were interviewed prior to and after the Great East Japan earthquake and tsunami found that having one’s home entirely destroyed was associated with a decline in household daily activities several years after the disaster (18). However, a study of older adults who were interviewed a year prior to and a year after Hurricane Sandy did not find that property damage was associated with functional outcomes (17). Mixed results from these studies suggest that the association between geographic proximity to the disaster and subsequent health is not well understood. Additional studies are needed to further clarify the predictive contribution of geographic proximity to health outcomes after a disaster.

Stress responses to disasters vary as a function of predisaster characteristics of survivors (43). Review studies reported that predisaster characteristics associated with postdisaster emotional or physical morbidity include a history of depression, type and number of chronic diseases, low social support, low physical functioning, and female sex (6, 40, 44). Many of these characteristics overlap with risks for onset of chronic diseases. Common risks for onset of chronic diseases include older age, obesity, comorbidities, depression, low levels of education, low levels of physical functioning, and low levels of social support (45–48). A better understanding of the impact of disaster exposure on the onset of chronic diseases in the years after disasters requires consideration of predisaster characteristics that could explain the association between older adults’ perceptions of their disaster-related psychosocial environmental stress and onset of chronic diseases.

## The Current Study

Our understanding of the impact of disaster exposure on the onset of chronic diseases is limited to studies that reveal higher hospital admissions in the weeks after a disaster. Prior studies have not considered whether disaster exposure influences the onset of chronic illnesses including CVD, diabetes, arthritis, and asthma. Using survey data from a large, state-wide sample of older people who were interviewed prior to and after Hurricane Sandy and linked Medicare data, we examine whether geographic proximity to the hurricane and reports of fear and distress during the hurricane are associated with new onset of chronic diseases after statistically controlling for predisaster risks for onset of chronic disease.

## Method

### Data and Sample

Data for this study are from 2 sources: self-reported data from the longitudinal ORANJ BOWL panel study and linked Medicare data. Detailed descriptions of the sampling methods that were used to achieve a representative sample of the population of community-living older adults from New Jersey are provided in prior publications (42, 49). We use self-reported data available at Waves 3 and 4 of the panel study. Hurricane Sandy occurred on October 29, 2012, which was approximately halfway between the Wave 3 data collection in 2011 (median date July 7, 2011) and the Wave 4 data collection in 2014 (median date January 24, 2014). In 2014, we mailed a questionnaire (Wave 4) to all ORANJ BOWL respondents known to be alive at Wave 3. We called participants who did not complete the interview, and whenever possible, we completed the interview by telephone.

We merged Medicare data with the self-report data through a secure process in which we sent the participant’s last name, sex, date of birth, and study identification number to the Centers for Medicare and Medicaid Services (CMS) where analysts identified beneficiaries who uniquely match the provided information. CMS data from matched participants used in this study were from the Medicare Beneficiary Summary Files and Chronic Conditions segment from January 1, 2013 through December 31, 2016.

**Figure 1.**
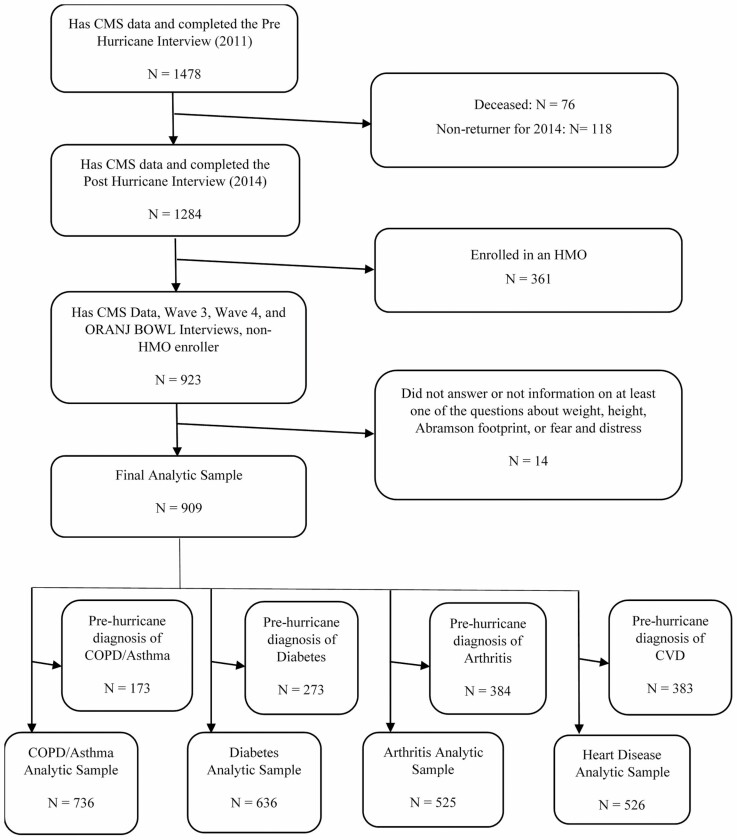
Derivation of the analytic samples. CMS = Centers for Medicare and Medicaid Services; COPD = chronic obstructive pulmonary disease; CVD = cardiovascular disease; HMO = Health Maintenance Organizations.

About 1 478 Medicare beneficiaries participated in the Wave 3 interview (Pre-Hurricane Interview). Of these, 76 died and 118 did not return for Wave 4 interview (Post-Hurricane Interview). Participants completing interviews both before and after Hurricane Sandy numbered 1 284. We excluded 361 participants enrolled in Health Maintenance Organizations (HMOs) because HMOs are not required to report specific episodes of utilization or the diagnoses associated with utilization. Of the remaining participants, we excluded 14 people from analyses because they did not answer at least one of the questions about demographic information, weight, height, or hurricane exposure. The final analytic sample consists of 909 participants (Figure 1). We did not detect any significant differences in characteristics between the original set of 1 478 participants and the final analytic sample. The average age of the original sample was 69.7 (*SD* = 4.9) compared to 70.0 (*SD* = 4.6) for the final analytic sample. The original sample consisted of 68% females versus 66.7% in the final analytic sample. In terms of reporting race, the percent of White in the original sample was 92% compared to 93.8% for the analytic sample.

Diagnosis dates for 4 chronic disease conditions derive from the Medicare data: (a) lung disease (including asthma or chronic obstructive pulmonary disease [COPD]), (b) diabetes, (c) arthritis (osteoarthritis or rheumatoid arthritis), and (d) CVD, including acute myocardial infarction, ischemic heart disease, congestive heart failure, stroke, or transient ischemic attack. We developed separate analytic samples for each of these 4 conditions by excluding participants already diagnosed with the disease(s) prior to Hurricane Sandy. Figure 1 displays a diagram of the derivation of the analytic samples for this study.

### Measures

#### Dependent variable

Our analyses focus on the onset of 4 chronic disease conditions: (a) lung disease, (b) diabetes, (c) arthritis, and (d) CVD. Using data from CMS, we developed 4 analytic samples to assess new onset of chronic diseases within each of the 4 chronic diseases. The lung disease analytic sample focused on the onset of COPD and asthma. It included 736 participants not diagnosed with COPD or asthma prior to the day Hurricane Sandy hit land. We developed a second analytic sample to assess onset of diabetes. That sample included 636 participants not diagnosed with diabetes prior to the hurricane. We developed a third analytic sample to assess onset of arthritis that included 525 participants not diagnosed with osteoarthritis or rheumatoid arthritis prior to the hurricane. We developed the final analytic sample to assess onset of CVD. That sample included 526 participants not diagnosed with acute myocardial infarction, ischemic heart disease, congestive heart failure, stroke, or transient ischemic attack prior to the day of the hurricane. We did not include hypertension in our definition of CVD because 60% of the sample had a diagnosis of hypertension prior to the hurricane, which would substantially limit the number of persons who could be included in the analytic sample for CVD. We conducted separate analyses for each of the 4 analytic samples (Figure 1).

#### Independent variables

Self-reported data provided the demographic information for the analyses, including sex and education level. Sex is a binary variable with male coded as 0 and female coded as 1. Self-reported education was categorized into a binary variable: 0 = no college education and 1 = some or more college education. We calculated age based on the return date of the Wave 3 interview. Using marital status information reported at Wave 3, we created a binary variable with 0 = married and 1 = nonmarried. We determined the presence of obesity by calculating respondents’ body mass index (BMI) from self-reports of weight and height obtained at the Wave 3 interview. The formula used to calculate BMI for weight measured in pounds and height in inches is $\frac{weight}{height^{2}}\times 703$. From the calculated BMI, we classified a BMI of under 30 as nonobese and coded as 0 while a BMI of 30 and over was classified as obese and coded as 1.

We used the Medicare beneficiary Chronic Conditions Data Warehouse to obtain a count of the number of chronic diseases diagnosed prior to the date of Hurricane Sandy. The chronic diseases included chronic kidney disease, COPD, asthma, diabetes, acute myocardial infarction, ischemic heart disease, congestive heart failure, stroke, transient ischemic attack, and any cancers (eg, breast, colorectal, prostate, lung, and endometrial). Each of the above chronic diseases diagnosed before the date of Hurricane Sandy is counted toward the total number of prehurricane morbidities. Diagnosis of depression prior to the hurricane is defined similarly and is coded as 1 for being diagnosed and 0 for lack of diagnosis. We did not include depression in the count of comorbidities because it is a known risk for onset of chronic diseases within the 4 types of chronic disease conditions considered in this study (48). Instead, we used the diagnosis of depression as a covariate in our modeling of onset of new chronic diseases.

We measured geographic exposure to the hurricane using the Abramson footprint (50) that identifies the regions hardest hit by the hurricane. The Abramson Footprint includes the 9 counties in New Jersey that the Federal Emergency Management Agency (FEMA) Modeling Task Force rated as having had “very high impact.” We coded people as living within the Abramson footprint (1) if they lived in a census block that (a) experienced a storm surge of at least one foot, (b) had at least 20% of their housing units destroyed or damaged according to FEMA assessments (minor or major), or (c) reported more than average numbers of valid FEMA housing assistance registrations. We coded people living in other areas as not in the footprint (0).

We assessed peritraumatic stress at Wave 4 which has been shown in prior research to be predictive of disaster outcomes including depression (41, 42) and poorer physical functioning (17). Also, this variable has been shown to predict greater use of emergency department admissions during the year after the disaster (12). Evidence of the convergence validity of the fear and distress variable is demonstrated by its strong association with PTSD (44, 51). For each of the 4 chronic condition analytic samples considered in this study, those who reported a lot of fear and distress were significantly more likely to meet criteria (52) for PTSD at significance levels of *p* < .01. We did not consider PTSD in this study due to insufficient power to detect associations with this variable. Less than 1% of those who did not report fear and distress and less than 21% of those who reported a lot of fear and distress met the criteria for PTSD for each of the 4 analytic cohorts (*p*s < .001). This variable was operationalized by asking participants, “Were you distressed or fearful during Hurricane Sandy?” Responses were “no” (coded 0), “a little” (coded 0) versus “a lot” (coded 1); the latter category reflected significant peritraumatic stress.

### Statistical Analyses

We computed descriptive statistics for the demographic and exposure variables and report means and standard deviations for continuous variables and percentage within each category for nominal variables for the final analytic sample as well as each of the data sample for each analytic sample associated with each type of chronic disease. We computed separate Cox proportional hazard models to examine the relationship between each of the disaster exposure variables, covariates, and the time from the day of Hurricane Sandy to onset of a new chronic disease within each of the 4 types of chronic disease conditions. Specifically, for the lung disease analytic sample, we determined time to onset of either COPD or asthma as the number of days between October 29, 2012 (the day of Hurricane Sandy) and the date the participant received diagnosis of either COPD or asthma. For participants who received diagnoses of both COPD and asthma, we defined time to onset as the date of the first diagnosis of either COPD or asthma. For the diabetes analytic sample, we determined time to onset of a diagnosis of diabetes. For the arthritis analytic sample, we determined time to onset of either osteoarthritis or rheumatoid arthritis, whichever came first. For the CVD analytic sample, we determined time to onset of the first diagnosis of the following diseases: acute myocardial infarction, ischemic heart disease, congestive heart failure, stroke, or transient ischemic attack. We had CMS data through December 31, 2016, so participants without a diagnosis of the chronic disease(s) included in a chronic disease condition are right-censored after December 31, 2016. Participants who died between Hurricane Sandy and December 31, 2016 were right-censored on the date of their death. We computed separate multivariable Cox proportional hazard models for each of the 4 categories of chronic disease conditions to determine whether hurricane exposure is associated with the time to onset of chronic disease conditions while controlling for characteristics known to be associated with onset of chronic diseases. We used SAS version 9.4 for all analyses.

## Results

### Characteristics of the Analytic Samples

Table 1 displays the sample characteristics for the total number of participants considered for inclusion in the 4 analytic samples as well as the sample characteristics for each analytic subsample associated with each of the 4 types of chronic disease conditions. Prior to the hurricane (Wave 3), participants were on average 70.0 years old. The average age of those included in the lung disease, diabetes, arthritis, and CVD analyses was slightly younger, ranging from 69.5 to 68.3. Although 67.7% of the total analytic sample was female, the percent of females was lower for the analytic sample for arthritis (58.5%). The percent of persons who had some college was 69.3% for those in the total sample, but slightly higher for the lung disease, diabetes, and the CVD analytic samples. The percent of those who were not married was 40.2% for the total sample, but was somewhat lower for the 4 analytic subsamples. The average number of comorbidities was 1.1 for the total sample, but lower for the analytic samples, especially the CVD analytic sample. The percent of the total analytic sample that met the criteria for obesity was 31.1%, but the percentage was lower for the 4 analytic samples. A diagnosis of depression was present for 16.2% of the total analytic sample, but the percentage was lower for the 4 analytic samples, especially for the arthritis analytic sample. Extreme hurricane exposure based on the Abraham footprint affected 10.1% of the total analytic sample; the rate was similar for each of the 4 analytic subsamples. Among the total analytic sample, 5.9% reported experiencing “a lot” of fear and distress, compared to 94.1% reporting “no” or “a little distress,” during the hurricane, with little difference in rates across the 4 analytic samples.

**Table 1.** Participant Characteristics for the Total Analytic Samples and for Each Chronic Disease Analytic Sample

Variable	Total Across Analytic Samples	Analytic Sample for Onset of COPD or Asthma	Analytic Sample for Onset of Diabetes	Analytic Sample for Onset of Arthritis	Analytic Sample for Onset of CVD
*N* ^+^ *	909	736	636	525	526
New onset of the chronic disease,%	—	12.09	14.47	32.00	31.48
Age, mean (*SD*)	70.05 (4.66)	69.50 (4.61)	69.27 (4.61)	68.38 (4.36)	68.24 (4.22)
Female, %	66.67	65.22	63.99	58.48	65.59
Some college,† %	69.31	71.33	71.86	69.33	72.43
Not married,‡ %	40.15	36.41	35.85	33.14	34.41
Number of comorbidities, mean (*SD*)	1.14 (1.27)	0.82 (1.00)	0.59 (0.85)	0.67 (0.97)	0.35 (0.63)
Obesity,§ %	31.13	27.31	23.27	25.71	26.81
Prehurricane diagnosis of depression,‖ %	16.17	14.13	11.01	8.57	9.70
High hurricane exposure,¶ %	10.12	9.51	9.91	8.95	10.08
Fear and distress,# %	5.94	6.11	5.19	4.76	5.51

*Note:* COPD = chronic obstructive pulmonary disease; CVD = cardiovascular disease.

*The total sample reflects all participants available for inclusion in the analytic samples for each category of chronic diseases. The analytic sample size differs for each chronic condition category because those with a diagnosis of a condition within a chronic condition category prior to Hurricane Sandy are not included in the analyses for that category.

^†^Those with some college were distinguished from those with no college or lower levels of education.

^‡^Not married were distinguished from those who stated they were married at the time of their interview. Not married includes those who were never married, divorced, separated, or widowed.

^§^Obesity was determined using the standard cutoff of a body mass index of ≥30 based on respondents’ prehurricane assessments of height and weight.

^‖^Prehurricane diagnosis of depression refers to those who were diagnosed with depression prior to the hurricane.

^¶^High hurricane exposure was measured as being in a census block with any of the following: a storm surge of ≥1 foot, ≥20% of houses damaged, more than the average number of FEMA housing registrations.

^#^Those who reported a lot of fear and distress from the hurricane were distinguished from those who reported some or none.

### Hazard of New Onset of Chronic Disease Conditions

Associations between each of the predictor variables and onset of each type of chronic disease condition (Table 2) show that older age, education, marital status, and depression were not associated with increased hazard for new onset of any of the 4 types of chronic disease conditions. Women had a smaller hazard than men for experiencing the onset of diabetes (*p* = .02) after the hurricane. Number of comorbidities prior to the hurricane was associated with increased hazard for onset of arthritis (*p* = .01) and CVD (*p* < .01) after the hurricane. Obesity was associated with increased hazard for new onset of diabetes (*p* < .01). Living within the Abramson footprint was not associated with onset of any of the 4 chronic disease conditions. Experiencing a lot of fear and distress during the hurricane significantly increased the hazard for the onset of lung disease (*p* < .01), diabetes (*p* < .01), and arthritis (*p* = .02).

**Table 2.** Association of Each Characteristic With Time to Onset of Chronic Diseases

Variable	COPD or Asthma	Diabetes	Arthritis	Heart Disease
	Hazard Ratio and 95% Confidence Interval			
Age	0.98 (0.94–1.03)	0.97 (0.92–1.01)	1.00 (0.97–1.04)	1.00 (0.96–1.05)
Female	1.12 (0.72–1.74)	0.61 (0.41–0.92)*	1.18 (0.86–1.61)	0.87 (0.59–1.27)
Some college	0.88 (0.56–1.37)	0.84 (0.54–1.30)	1.33 (0.94–1.88)	0.88 (0.59–1.31)
Not married	1.14 (0.75–1.75)	1.06 (0.69–1.61)	1.18 (0.86–1.61)	0.88 (0.59–1.31)
Number of comorbidities	1.18 (0.98–1.43)	1.073 (0.85–1.36)	1.20 (1.04–1.38)*	1.48 (1.16–1.90)**
Obesity	1.46 (0.95–2.26)	**2.27 (1.57**–3.61)***	1.07 (0.76–1.51)	1.44 (0.98–2.13)
Prehurricane diagnosis of depression	0.95 (0.52–1.75)	0.99 (0.52–1.92)	1.54 (0.95–2.48)	0.99 (0.53–1.84)
High hurricane exposure	1.55 (0.84–2.84)	1.12 (0.58–2.15)	1.09 (0.61–1.82)	0.93 (0.50–1.73)
Fear and distress	2.67 (1.45–4.91)**	2.53 (1.31–4.87)**	2.01 (1.14–3.54)*	1.44 (0.70–2.96)

*Note:* COPD = chronic obstructive pulmonary disease.

*.01 < *p* ≤ .05, **.001 < *p* ≤ .01, ***p ≤ .001.

Table 3 presents the results from the multivariable models for each chronic disease condition. After statistically controlling for common risks for experiencing stress after a disaster, and common risks for onset of chronic diseases, experiencing a lot of fear and distress during the hurricane was associated with approximately a twofold increase in the hazard of acquiring new onset of COPD or asthma (*p* = .01), diabetes (*p* < .01), and arthritis (*p* = .02), but not new onset of CVD (Table 3).

**Table 3.** Multivariable Cox Proportional Hazard Models for Time to Onset of Chronic Diseases

Variable	Chronic Condition			
	COPD or Asthma	Diabetes	Arthritis	Heart Disease
	Hazard Ratio and 95% Confidence Interval			
Age	0.96 (0.91–1.01)	0.96 (0.91–1.02)	0.99 (0.95–1.03)	0.99 (0.94–1.04)
Female	1.06 (0.66–1.68)	0.57 (0.37–0.88)*	1.15 (0.83–1.58)	0.84 (0.57–1.26)
Some college	0.87 (0.55–1.37)	0.84 (0.54–1.31)	1.40 (0.998–1.99)	0.89 (0.59–1.33)
Not married	1.13 (0.72–1.77)	1.24 (0.79–1.94)	1.19 (0.86–1.65)	0.89 (0.59–1.34)
Prehurricane diagnosis of depression	0.83 (0.44–1.57)	1.03 (0.52–2.05)	1.33 (0.80–2.20)	0.83 (0.44–1.58)
Number of comorbidities	1.24 (1.00–1.54)	1.17 (0.90–1.48)	**1.22 (1.04**–1.43)**	1.55 (1.19–2.02)**
Obesity	1.35 (0.86–2.10)	2.29 (1.50–3.50)***	0.98 (0.69–1.40)	1.34 (0.90–1.99)
High hurricane exposure	1.41 (0.75–2.64)	1.11 (0.57–2.18)	1.04 (0.61–1.77)	0.97 (0.51–1.82)
Fear and distress	2.29 (1.21–4.33)**	**2.96 (1.50**–5.84)**	2.02 (1.13–3.62)*	1.41 (0.68–2.92)

*Note:* COPD = chronic obstructive pulmonary disease.

*0.01 < *p* ≤ .05, **.001 < *p* ≤ .01, ***p ≤ .001.

## Discussion and Implications

Community-dwelling older people who reported experiencing high levels of fear and distress during Hurricane Sandy had more than twice the risk of developing lung disease, diabetes, and arthritis than people who did not experience fear and distress. Reports of fear and distress contributed predictive value beyond well-known risks for onset of chronic diseases including older age, education, marital status, number of comorbidities, obesity, and depression. The findings reveal that the health effects of disaster exposure extend beyond the weeks and months after a disaster and include onset of chronic diseases that are associated with loss of functioning and early mortality (53, 54). To our knowledge, this is the first study to show the long-term impact of peritraumatic stress on onset of chronic disease conditions.

This study focused on older adults due to their inherent vulnerability for acquiring new onset of chronic diseases. In the context of disasters, some older adults’ have additional vulnerability because they are not prepared for negotiating the effects of a disaster. The Health and Retirement Survey, a national panel study of adults aged 50 and older, deployed a disaster survey in 2010 (55). Study findings revealed that disaster preparedness was lowest among people aged 80 and older. For example, those 80 and older were significantly less likely to (a) have a means by which to receive communication during a disaster, (b) live in a household with a car and a driver, or (c) have knowledge of a shelter in the community. In addition, compared to middle-aged adults, those aged 65 and older were more than twice as likely to have mobility limitations that could reduce their ability to immediately leave the site of a disaster. The existing health conditions of older adults further increase their vulnerability during a disaster due to disaster-related interruptions in medications, needed medical devices, nutrition, and water (56, 57). Lack of preparedness in combination with the vulnerability of coping with health and functional needs during the disaster may have contributed to some older adults’ fear and distress from the disaster, although we did not capture information about disaster preparedness, access to transportation, medical, food, and water resources immediately after the hurricane. Nonetheless, our analyses find that 6% of respondents reported they experienced a lot of fear and distress during the hurricane, which increased their risk for future onset of chronic conditions.

Previous studies found that demographic, health, and psychosocial factors including older age, sex, education, marital status, obesity, depression, and prior chronic diseases increase the risk for onset of chronic diseases (45, 58, 59). In contrast, we did not find that age, education, marital status, or prior history of depression were associated with onset of chronic diseases in the 4 years after the hurricane. There are several explanations for the lack of associations between common risks and onset of chronic diseases found in this study. First, the interval during which we assessed time to onset of new conditions was only 4 years, which is a much shorter interval than earlier studies of onset of chronic conditions (58, 59). Second, in contrast with prior studies that included much younger adults, we assessed new onset of chronic diseases in older adults whose average age was 70, an age by which the effects of early and midlife risks may have already contributed to onset of the chronic disease conditions considered in this study. In fact, 19% of the total sample available for analyses had a diagnosis of lung disease (asthma or COPD) prior to the hurricane, 30% had a prior diagnosis of diabetes, 42% had a prior diagnosis of arthritis, and 42% had a prior diagnosis of CVD. Third, eliminating those with a prior diagnosis resulted in analytic samples that were healthier than the total sample from which the chronic disease analytic samples were drawn. Specifically, the analytic samples had fewer chronic diseases, and fewer persons with obesity and depression compared to the total sample. The findings show that compared to well-known risks for onset of chronic diseases, experiencing a lot of fear and distress from the hurricane was the most salient risk for onset of chronic diseases in the years after Hurricane Sandy.

The findings from this study provide evidence that a single-item assessment of experiencing a lot of fear and distress during the hurricane is sensitive to detecting risk for onset of chronic diseases and in the years after a disaster. The findings have direct relevance for a clinical practice where single-item health assessments are regularly used to provide quick assessments of patients’ risk for poor health outcomes (60, 61). Querying patients about whether they experienced significant fear and distress after a disaster provides the opportunity to discuss patients’ future risk for onset of chronic diseases that commonly develop in later life. Prevalence of having 2 or more chronic diseases increases 50% between the sixth and seventh decades of life (62). Given that multiple chronic diseases increase older adults’ risk for future functional decline (21–24), early mortality (9), and higher Medicare expenditures (63), it is critical to develop clinically feasible tools for determining those who would benefit from health monitoring and preventive interventions to reduce onset of chronic diseases.

On the other hand, when disaster exposure was operationalized using geographic proximity to the hurricane, we did not find that disaster exposure was associated with the onset of chronic diseases. These findings are similar to those reported by a prior study of the effects of Hurricane Sandy on trajectories of functional decline (17) and may be a function of a geographic location being too crude of an exposure indicator. As is true for other hurricanes, there was some randomness to the path taken by Hurricane Sandy. Homes within just a few blocks of one another sustained very different effects, with some homes destroyed while others bore no damage. Finding that peritraumatic stress experienced during a hurricane is associated with poor health outcomes, but that geographic proximity to the storm is not, has policy implications for postdisaster care needs of older adults. In a discussion of mental health consequences of disasters, Goldmann and Galea (38) provided guidelines for postdisaster interventions to reduce disaster-related stress reactions. A first priority is meeting basic needs for food, shelter, and medical supplies. Second, it is important to address modifiable stressors so that victims can return to their predisaster routines as quickly as possible. Third, in the postdisaster period, it is important to identify the risk for developing psychopathology. These assessments help identify people who would benefit from treating emerging symptoms with the goal of preventing emerging psychiatric illness. Although a minority of disaster victims develop psychiatric illness after a disaster, many experience some form of psychological distress (64). Findings from this study provide evidence that it is important to address postdisaster psychological symptoms even among those victims whose symptoms do not meet diagnostic criteria for major psychiatric illness.

This study was not designed to assess the mechanisms by which experiencing fear and distress leads to the future onset of chronic diseases. However, the premise underlying this study is that unexpected threats associated with a natural disaster can lead to perceived stress, which in turn can induce physiologic responses. Others have shown that sudden, unexpected, threats from a natural disaster can induce stress (44, 65), and that disaster exposure can lead to physiologic responses including increases in heart rates, systolic blood pressure, serum cholesterol, and triglycerides soon after a natural disaster (36, 66). Both individual-level factors (eg, perceived threat from the hurricane) and behavioral factors can moderate physiologic responses to the perceived stress (67). We did not measure variation in behavioral responses to the hurricane, but others have found that disaster exposure can lead to a decline in health behaviors including decreased physical activity and weight gain (68). The strong association between the prehurricane assessment of obesity and onset of diabetes may reflect diet and exercise behaviors that continued after the disaster. Although prior studies have demonstrated the association between stress and onset of chronic diseases, to our knowledge, this is the first study to show that disaster-related stress increases the risk for chronic disease conditions among older adults.

Strengths of the study come from the use of linked Medicare and survey data. Longitudinal data collected before and after the hurricane are unique, enabling understanding of the directionality of effects. Furthermore, because disaster exposure is an unplanned event, the effects of self-selection on study outcomes are minimized compared to cross-sectional studies of onset of chronic diseases (69). Unlike prior studies that relied on self-reported data to determine the onset of a chronic condition, the use of Medicare data allowed us to determine the time to onset of chronic diseases after the hurricane, increasing the statistical power to detect an association between assessments of fear and distress and onset of chronic diseases.

However, as is true of all studies, this one has its limitations. First, although the sample size was sufficiently large to detect moderate to large effect sizes, it was not large enough to detect smaller effect sizes. For example, living in an area of high hurricane exposure was associated with a 50% increase in the hazard of new onset of lung disease, but the estimate was not statistically significant. Second, the generalizability of findings is limited to older Medicare beneficiaries exposed to a hurricane. It is possible that risks and incidence of new chronic disease are different for other types of disasters such as earthquakes, tornados, and wildfires. Third, while it is possible that people experienced disruptions to their medical care in the weeks after the hurricane, we do not have access to this information. Fourth, we do not have information about the causes or timing of death for persons who did not return for the Wave 4 interviews. Fifth, we did not assess factors that may have contributed to chronic disease during the 4-year interval during which we assessed onset of chronic illnesses. A better understanding of postdisaster events or behaviors that modify the associations between fear and distress from the disaster and onset of chronic diseases would inform prevention interventions. Until such studies are conducted, clinicians should be aware of the prognostic significance of experiencing a lot of fear and distress from a disaster, with the goal of monitoring disaster victims’ mental and physical health with the goal of preventing poor health outcomes.
